# Statement of retraction: MicroRNA-409 regulates the proliferation and invasion of breast cancer cell lines by targeting special AT-rich sequence-binding protein 1 (SATB1)

**DOI:** 10.1080/21655979.2026.2730805

**Published:** 2026-10-01

We, the Editors and Publisher of the journal *Bioengineered*, have retracted the following article from publication.

Chen, Z., Sang, M. X., Geng, C. Z., & Jia, H. Q. (2022). MicroRNA-409 regulates the proliferation and invasion of breast cancer cell lines by targeting special AT-rich sequence-binding protein 1 (SATB1). *Bioengineered*, *13*(5), 13045–13054. https://doi.org/10.1080/21655979.2022.2073320 

Since publication, significant concerns have been raised about the integrity of the data and reported results in the article.

When approached for an explanation, the authors have not responded to our queries and so these serious concerns remain unaddressed.  

As verifying the validity of published work is core to the integrity of the scholarly record, we are therefore retracting the article. The authors have been informed of this decision.

We have been informed in our decision-making by our policy on publishing ethics and integrity and COPE guidelines.  

The retracted article will remain online to maintain the scholarly record and will be digitally watermarked on each page as ‘Retracted’.  

